# Sickness certification as a complex professional and collaborative activity - a qualitative study

**DOI:** 10.1186/1471-2458-12-702

**Published:** 2012-08-28

**Authors:** Anna Kiessling, Britt Arrelöv

**Affiliations:** 1 Department of Clinical Sciences, Danderyd Hospital, Karolinska Institutet, 182 88, Stockholm, Sweden; 2 Stockholm County Council, Stockholm, Sweden; 3 Medical Management Center, Department of Learning, Informatics, Management and Ethics, Karolinska Institutet, Stockholm, Sweden

## Abstract

**Background:**

Physicians have an important but problematic task to issue sickness certifications. A manifold of studies have identified a wide spectrum of medical and insurance-related problems in sickness certification. Despite educational efforts aiming to improve physicians’ knowledge of social insurance medicine there are no signs of reduction of these problems. We hypothesised that the quality deficits is not only due to lack of knowledge among issuing physicians. The aim of the study was to explore physicians’ challenges when handling sickness certification in relation to their professional roles as physicians and to their interaction with different stakeholders.

**Methods:**

One hundred seventy-seven physicians in Stockholm County, Sweden, participated in a sick-listing audit program. Participants identified challenges in handling sick-leave issues and formulated action plans for improvement. Challenges and responsible stakeholders were identified in the action plans. To deepen the understanding facilitators of the program were interviewed. A qualitative content analysis was performed exploring challenge categories and categories of stakeholders with responsibility to initiate actions to improve the quality of the sick-listing process. The challenge categories were then related by their content to professional competence roles in accord with the Canadian Medical Education Directions for Specialists (CanMEDS) framework and to the stakeholder categories.

**Results:**

Seven categories of challenges were identified. Practitioner patient interaction, Work capacity assessment, Interaction with the Social Insurance Administration, The patient’s workplace and the labour market, Sick-listing practice, Collaboration and resource allocation within the Health Care System, Leadership and routines at the Health Care Unit. The challenges were related to all seven CanMEDS roles. Five categories of stakeholders were identified and several stakeholders were involved in each challenge category.

**Conclusions:**

Physicians performing sickness certification tasks experience a complex variety of challenges. From physician perspective actions to handle these need to be initiated in interaction with both medical and non-medical stakeholders. The relation between the challenges and a well-established professional competence framework revealed a complex pattern. Thus, from a public health perspective, educational activities aimed to improve the sick-listing process should address all physician competences including identification and interaction with stakeholders, and not just knowledge of social insurance medicine.

## Background

Clinical decision-making is a complex task [1,2]. When it also includes sick-listing decisions it becomes even more complex. In these cases the physician has to evaluate the patients’ medical problem including an assessment of function and work capacity. Based on this assessment the physician issues a medical certificate with a prediction of the recovery of the patient’s work capacity [3,4]. Few physicians perceive that they have enough knowledge of the demand of the patient’s workplace, the labour market, or the social insurance legislation in order to fulfil the task. Since decades physicians have reported negative feelings and loyalty conflicts in relation to their sick-listing duties [5-10].

Swedish physicians reported a variety of problems in two questionnaire surveys carried out 2004 and 2008. Problems reported were related to handling disagreements with patients about the need of a sickness certification; decisions of whether to certify a prolongation of a sick-leave period that was initiated by another physician; assessments of a patient’s work ability; and determination of optimal duration and degree of certification [11-13]. Several other studies in different countries have reported similar findings [14-20].

In most western countries physicians have an important but complex role as gatekeeper of the Social Insurance system, where illness and disability are used as eligibility criteria of sickness benefits [21,22]. However, there are no golden standard in how to assess disability and countries have different social insurance legislations and regulations of medical certification of incapacity to work [23]. In Sweden a medical certificate issued by a physician is needed after seven days of self-certification. Thereby sick-listing is a regular task of many Swedish physicians [11,13]. The sick listed person’s employer decide about benefits during the first 14 days, thereafter and for unemployed persons the Social Insurance Administration (SIA) make the decisions.

As a certifier of a medical certificate, the physician decides whether a patient might apply for a benefit or not. The physician has to balance the claim of the individual with the formal rules of society [21]. The quality of content in the medical certificates has been questioned in various studies [23-25]. Taken together a manifold of studies have identified a wide spectrum of medical and insurance-related problems in sickness certification.

In order to improve the sickness certification process in Sweden several educational activities directed towards physicians have been offered by the SIA. The learning activities have been traditionally organized with lectures and written material, at least in Sweden. The purpose has been to improve physicians’ handling of sick-listing by means of a better knowledge in social insurance medicine and in how to issue medical certificates. However physician’s performance in these complex situations depends on a variety of factors. In a study by Swartling et al barriers to good sick listing practice could be identified both within and outside of the healthcare system [19]. Thereby physicians’ work with sick-listing can not be seen isolated at individual level but in interaction with several both medical and non medical stakeholders.

Professional physician roles and competencies can be described based on different but partly overlapping theoretical frameworks. In this study we have analysed and discussed physicians’ problems with sick-listing in relation to the CanMEDS Roles Framework [26]. CanMEDS states seven professional roles that a physician has to fulfil. The framework state *Medical Expert* as the central role including diagnostic and therapeutic skills for ethical and effective patient care, to access and apply relevant information to clinical practice and provide effective consultation services with respect to patient care, education and legal opinions. The role *Communicator* includes to establish therapeutic relationship with patients/families, to obtain and synthesize relevant history from patients/families/communities, to listen effectively and to discuss appropriate information with patients/families and the health care team. The role *Collaborator* implies to consult effectively with other physicians and health care professionals and to contribute effectively to other interdisciplinary team activities. As a *Manager* physicians have to utilize resources effectively to balance patient care, learning needs, and outside activities, to allocate finite health care resources wisely, to work effectively and efficiently in a health care organization and to utilize information technology to optimize patient care, life-long learning and other activities. As a *Health Advocate* physicians have to identify the important determinants of health affecting patients, to contribute effectively to improved health of patients and communities and to recognize and respond to those issues where advocacy is appropriate. The role as a *Scholar* implies to develop, implement and monitor a personal continuing education strategy, to critically appraise sources of medical information, to facilitate learning of patients, house staff/students and other health professionals and to contribute to development of new knowledge. Finally the role as a *Professional* implies to deliver highest quality care with integrity, honesty and compassion, to exhibit appropriate personal and interpersonal professional behaviours and to practise medicine ethically consistent with obligations of a physician. The seven roles are not separated but complementary and partly overlapping. The physician is supposed to learn lifelong by continuous and systematic development of all above stated competencies. No studies have hitherto tried to understand physicians’ challenges with sickness certification in relation to a competency framework.

The aim of the study was to explore physicians’ challenges when handling sickness certification and to relate those to professional roles and to physicians’ interactions with different stakeholders.

## Methods

### Study population

One hundred seventy-seven physicians working in Stockholm, Sweden, participated in an audit aimed to improve the sick-listing process during 2006-2007. One hundred fifty-six of the participants were general practitioners and 21 were specialists in rehabilitation medicine, gynaecology, internal medicine and surgery.

In the first step participants registered a consecutive sample of patient encounters including sick-listing decisions during a three-week period. They registered in total 1380 patient encounters concerning sickness absence considerations. The registration was performed on a web-based questionnaire with questions concerning sick-listing and if the physician perceived the sick-listing as problematic. If so they also graded and indicated the type/-s of problems perceived.

In the second step group meetings were arranged in order to discuss the results from the registered patient cases. Well experienced physicians with special education and work experience as medical advisors of the SIA acted as facilitators in the group-meetings. Physicians participated in one or two group meetings together with colleagues at their own clinical department. How many that participated in each meeting depended on the number of physicians working in the different departments (3-10). During the meetings the over-all results of the web-questionnaire were presented and participants discussed and compared these results with their own results on the web-questionnaire. During the meeting they could also bring up previous experiences of sick-listing challenges. They filled in a protocol on which they specified the challenges noticed.

In a third step the participants wrote action plans for improvement of the sick-listing process. The action plans were based on the protocol with challenges formulated at the group-meeting. The plans were written on a standardised form with predefined headings (definition of challenge; actions to be taken; outcome measures; time tables). Several challenges could be listed in each plan. Participants could either formulate an individual action plan or collaborate with colleagues. Most of them chose to collaborate and formulate one action plan for the entire department.

### Data collection

A copy of each action plan was sent to the project's administrative office. Data was collected from 37 plans.

Data was further collected from two group interviews with the facilitators. The interviews were conducted in order to get a richer data base and a deeper understanding of the challenge areas perceived by the physicians’. Four facilitators participated in each interview. A moderator, the second author, and an assistant moderator led the group interviews. The role of the moderator was to open the discussion, to stress the confidentiality between participants, to listen, to encourage, to shape and to round off the discussion. The role of the assistant moderator was to observe, to take notes and to ask additional questions [27]. Each interview was opened by the question ‘Please tell me about challenges with sick-listing discussed among the physicians during the small group meeting? With the aim in mind, probes tended to be open-ended or specific to the participants’ comments, such as ‘Can you tell me more specific about it?’ The group interviews were tape-recorded.

### Data analysis

The action plans were scrutinized systematically. All content including statement about challenges with sick-listing formed one unit of analysis, and all statements including suggested stakeholders responsible to act formed another unit of analysis. Stakeholders in this study were defined as the person, persons or organisation having the main responsibility to initiate handling of a challenge. The stakeholder could either be explicitly stated in the action plan or become obvious, based on the description of the problem and the suggested actions.

We performed an inductive qualitative content analysis [28]. Condensed meaning units were identified for all challenges respectively for all stakeholders. Condensed meaning units were labelled with codes and codes with a related content were subsequently grouped together and preliminary categories were formulated.

The recorded group interviews were then listened through and transcribed verbatim. The text was read several times to comprehend a sense of the whole.

Meaning units, i.e. strings of words, a sentence or several sentences bound together by their content were identified. Condensed meaning units with a related content were grouped together. The grouping of interview data was systematically compared with the preliminary challenge categories formed of the data from the plans. After discussion between the researchers consensus was reached regarding the categorisation and labelling of all data.

After that the challenge categories were related by their content to the seven professional competence roles according to CanMEDS 2005 Physician Competency framework [26]: Medical Expert (central competence), Communicator, Collaborator, Manager, Health Advocate, Scholar and Professional.

In order to evaluate the accountability of the qualitative results the number of challenges in each category was counted together with the number of corresponding responsible stakeholder categories. An example of the analytical steps is shown in Table 1.

**Table 1:** The analytical steps

Meaning unit	It is a misuse of recourses. Instead of one visit at the specialist clinic, the patient first has to see the specialist for a diagnose and then has to make an additional appointment to the family doctor for the medical certificate – and that for the same disease
Condensed meaning unit	A misuse of resources. First visit a specialist for a diagnose and then the family doctor for the medical certificate
Code	Low effectiveness of the use of limited health care resources
Challenge category	Collaboration and resource allocation within the healthcare system
Associated generic competence*	Contribute effectively to improved health of patients and communities and to recognize and respond to those issues where advocacy is appropriate
Physician CanMEDS role	Health advocate
Stakeholders involved	The Health Care System of the region

Some quotes were used to illustrate the results and to validate the categorization [29]. Certain linguistic and grammatical revisions have been carried out because of the transition between spoken and written language and when text data were translated into English [30].

The study conforms to the principles outlined in the Declaration of Helsinki1975, revised in Hong Kong 1989. Written informed consent was obtained from department chiefs prior to acceptance of participation in the audit and from participating physicians in the web-based questionnaire and from facilitators prior to acceptance to participate in an interview after the group meeting. The Regional Ethical Review Board in Stockholm, Sweden, approved the study.

## Results

Each action plan contained from one up to ten challenges, with a median of three. A total of 178 challenges were reported in the plans. Stated challenges formed seven categories as described below. Five categories of stakeholders responsible to initiate actions were identified: The physician in charge, the concerned Health Care Unit, the Health Care System of the region, the patient and the employer, the Social Insurance Administration and legislation. As shown in Table 2 there were several stakeholders involved in each challenge category.

**Table 2:** Number of challenges stated in the plans by group of stakeholders responsible to initiate action

**Problem category**	**Stakeholders responsible for handling the challenges**					
	**All**	**Physician**	**HCU**^**a**^	**HCS**^**b**^	**Patient/ employer**	**SIA**^**c**^
All stated challenges^d^	178 (100)	80 (45)	28 (15)	17 (10)	24 (14)	29 (16)
Practitioner and patient interaction	33	23	7		3	
Work capacity assessment	27	25	2			
Interaction with the Social Insurance Administration	47	12	2		5	28
The patient’s workplace and the labour market	14	2	3		8	1
Sick-listing practice	12	6	1		5	
Cooperation and resource allocation within the Health Care System	29	7	3	17	2	
Leadership and routines at the Health Care Unit	16	5	10		1	

^a^HCU=The management of concerned Health Care Unit.

^b^HCS=The Health Care System in the region.

^c^SIA=The Social Insurance Administration and legislation system.

^d^Number of challenges stated in the action plans; given as Number (%).

The group interview data comprised a variety of challenges with sick-listing. These challenges were in agreement with the challenges identified in the action plans. The challenges with sick-listing were connected to generic competences needed to fulfil all seven professional roles as defined by CanMEDS. All challenge categories needed competencies from at least two roles in their handling.

### Practitioner patient interaction

This category illustrates the physicians’ perceptions of challenges related to their handling of the individual patient contact. To handle challenges in this category the physician had to integrate competencies as a **Professional** and as a **Communicator**. Both action plans and interview data included descriptions of difficulties and frustration to deliver highest quality care with integrity, honesty and compassion when handling situations when the patient’s demand of a medical certificate and the judgement of the physician were in conflict.

"“When the physician and the patient had different opinions regarding the need of sick leave – patients just pass by and seek another colleague to get the certificate”"

Furthermore difficulties were described in the communication with patients and in establishing a therapeutic relationship with patients motivating them to participate in a rehabilitation programme. Physicians experienced that it was more time consuming to motivate a patient to work than to issue a sickness certificate – and they had difficulties to manage this when they had no time in their daily schedule.

"“The patient was supposed to pass out through the door – she stopped and asked for a sick leave certificate. That question could spoil all plans of the day and the working schedule would be delayed. This resulted in that the physician gave up his code of conduct and issued the certificate without further argument”"

It was perceived as difficult to practise medicine ethically consistent with obligations and avoid unnecessary sick-listing, particularly when time was a restriction.

"“…The conflict… the double roles of a physician to both be a person executing public authority duties and (at the same time) be a responsible assisting physician …”"

Interviews statements indicated feelings of loneliness, sleeping difficulties and shame when the physician felt unprofessional in the handling of sick-listing issues.

"“They (the physicians) felt guilty and that they had not done enough…”"

### Work capacity assessment

The category illustrates the physicians’ view on difficulties to assess work capacity. To handle the challenges of this category the physician had to use competencies as a **Medical expert, Communicator** and **Scholar**. A lot of challenges and ambiguity were stated regarding work capacity assessment in general, its definition, and how it related to the medical history. Physicians expressed a lack of competence in how to access and apply knowledge regarding the demands of the patient’s work place and integrate it with how the patient’s disease/-s could afflict function in general and the patient’s ability to perform his or her work in particular.

"“In patients with psychiatric diagnoses – physicians described challenges with the assessment of work capacity”"

In communication with patients it was perceived difficult to handle a sick-listing discussion with unemployed patients. Further to pose effective questions to patients about their work conditions and disabilities. As a scholar a struggle was to maintain lifelong learning, to be updated and to follow all new regulations instead of continuing with old routines.

"“The older physicians did not oppose (the patients’ will) and had a more comprehensive perspective than younger physicians – they (the younger) had just learnt how it was meant to be done (the regulations)”"

"“…They (the physicians) had a lack of knowledge regarding social insurance formal rules and regulations… “"

### Interaction with the Social insurance administration

This category illustrates the physicians’ struggling to establish an efficient interaction with personnel at the Social insurance administration. Challenges in this category were well matched with the competence role as **Collaborator** but challenges also involved difficulties in the role as a **Scholar**. Challenges when the Social Insurance Administration (SIA) questioned medical certificates were recurrently discussed. It was perceived challenging to formulate sickness certificates in medically complicated patient cases and subsequently get the SIO to understand the situation. Slow handling by clerks and frequent exchange of clerks at the SIA was also perceived as a challenge.

"“If I wanted to help my patient with work place training, it could take half a year to get permit (by the SIA) to initiate it…”"

Physicians expressed difficulties to keep up with their own life-long learning in social insurance medicine. Furthermore, they struggled with how they could facilitate the learning of the Social Insurance Officers (SIO) in the understanding of medicine.

### The patient, the workplace and the labour market

This category illustrates the physicians’ effort to handle patient cases where it was needed to understand the patient’s medical problem and also his or her workplace, and or the demands at the labour market and how to interact with employers. Physicians used mainly **Collaborator** and **Communicator** competences to solve challenges of this category. Workplace related conflicts that resulted in sick-listing were perceived as problematic. To get in contact with the employer was a time consuming process and sometimes without any prospect of success.

"“It was difficult to initiate a dialogue together with the employer and the patient”"

Disability, illiteracy and e.g. language difficulties among immigrants were perceived as challenging in relation to the labour market. It was difficult to motivate patients who had been habituated to be on sick leave to attend rehabilitation programs.

### Sick-listing practice

This category illustrates how physicians struggled to find guidance in a structure or standard to handle complicated sick-listing issues. **Medical Expert** and **Health Advocate** are obvious roles in relation to this category. As a medical expert, competence to provide effective consultation services with respect to patient care and to legal opinions was challenging. A great ambiguity regarding sickness certification practice in relation to certain diseases was identified. In spite of national normative standards for sick leave lengths for common diagnoses there were several examples in the data illustrating challenges related to insufficient guidance in the sick-listing decision. Two challenging situations mentioned were sick-listing during pregnancy, and how to response to individual patients’ health needs in relation to the disease and its prognosis.

"“ Burn out - especially in women at executive positions on sick leave nothing could be done to help…”"

As a health advocate it was challenging to assess long-term effects of a decision to issue a medical certificate as compared to a refusal. It was unclear to them, which decision would promote the patient’s health in the long run. To identify opportunities to health promotion and to prevent disease of certain diagnoses were difficult. It was also recognised as difficult to distinguish between stress-related problems and depression.

### Collaboration and resource allocation within the Healthcare system

This category to illustrates physicians’ dependence of a well functioning collaboration between different departments in the health care network. Competences as both **Collaborator** and **Health advocate** were needed in order to handle challenges of this category. The waiting time for medical examination by specialists was perceived as a challenge. Lack of specific competence resources leading to unacceptably long waiting lists was also important challenges e.g. to visit a specialist in neuropsychiatry, a psychologist or the SIO. As a health advocate physicians had difficulties to support routines perceived as a misuse of resources. The general practitioner perceived that it as problematic to continue to handle sick leave issue when a patient was referred to a specialist in secondary care. Furthermore they frequently felt that the information from the specialist was insufficient.

"“Investigation for back pain as an example, if you send a patient for consultation to an orthopaedic specialist – afterwards the patient contacts you to evaluate the need of sick leave, but you have as yet not received the expert opinion from the specialist”"

### Leadership and routines at the Healthcare unit

This category illustrates how a dysfunctional leadership and management at the workplace could cause challenges for the physician handling sick-listing issues. Challenges of this category were related to the competence roles as **Manager** and **Collaborator**. Several statements were identified tapping different aspects of the organisation at the unit and of ambiguities regarding responsibilities of various professions.

"“It was different depending on leadership, attitude and culture at the workplace – it influenced quality”"

"“Who is responsible for decisions regarding patient care? If the patient sees the social welfare officer assessing that the patient is in need of sick leave for a certain time, then the physician is supposed to use this assessment and effectuate it in a sickness certificate…”"

To collaborate, as a team was difficult if it was an uneven distribution of workload between physicians, a high turnover of physicians, increased workload and deficient local routines and policies regarding sick listing.

"“Some (physicians) had a heavier workload with more patients with socio-economic challenges. It depended a lot on the commission for the out-patient clinic and the population living in the area”"

Physicians also pointed out a lack of time for reflection together with colleagues. The patients’ increased opportunities to choose a specific physician led to challenges in some cases when patients wanted to change physician due to different opinions as regards sick-listing.

"“If the physician did not agree with the patient regarding the need of sick leave the patient changed physician in order to receive a sickness certificate”"

**Figure 1:**
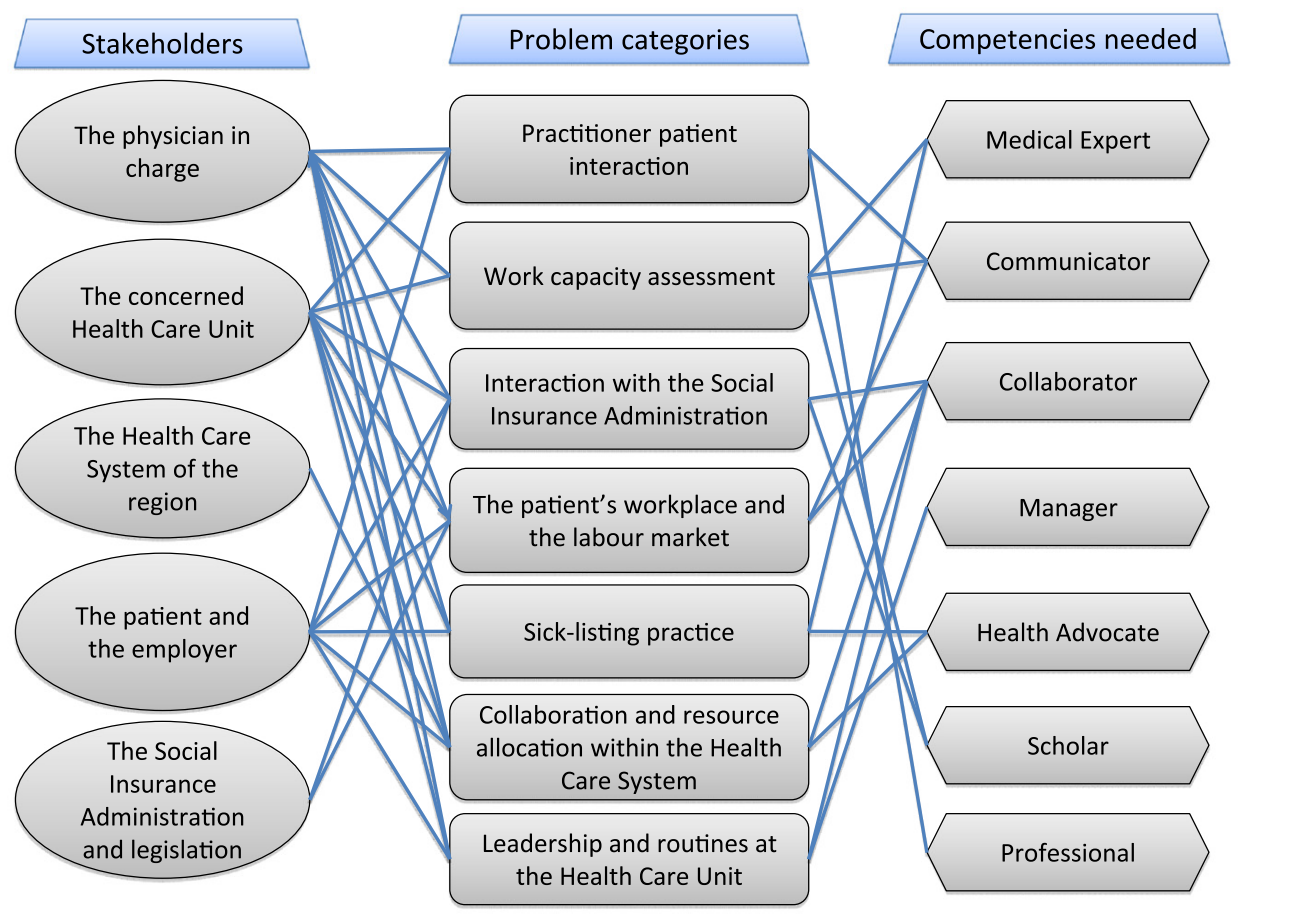
**Key stakeholders responsible to initiate action to solve different challenge categories when handling sickness certification issues and associated physician competence roles.** The complex pattern of challenges perceived by physicians in handling of sickness certification issues. Physicians have to handle the challenge categories shown in the middle in interaction with the responsible stakeholders listed to the left and by using the professional competence roles according to CanMEDs [26] listed to the right.

The Figure 1 illustrates the multiple connections between the challenge categories, the competence roles and the responsible stakeholders.

## Discussion

The participating physicians stated a wide variety of challenges regarding sick listing. The study confirmed that sick listing is a very complex task and that physicians have to interact with others and need to use a combination of professional competences [26] to handle it properly. In sick listing the physician have to simultaneously act in double roles, both as the individual patient’s advocate and as health advocate of society [10,31,32].

A combination of actions was almost always proposed to handle the entire challenging situation as described by the participants. The results point out the need for interaction with several stakeholders representing different cultures. In a Swiss study of sickness certification in primary care the authors stated that the certification process should be improved through better coordination and communication between all involved parties [15].

A similar range of challenges in sick-listing activities has been reported in other studies [9,11-14,19]. Challenges are well known as regards work capacity assessment in short and long term, sick-listing in certain complex medical and psychosocial situations and when patient demand of a medical certificate and the judgement of a physician are in conflict. Previously, situations have been identified as challenges when collaboration with other medical or non-medical parties is mandatory. However, this study points out the physician as one actor in a multiprofessional pattern of several actors, rather than a solely decision maker interacting with the individual patient. As in line with previous studies we have shown deficiencies in the organisation and a managerial shortage of the Swedish health care system as regards handling of sick-listing patients [14,19,33].

Swedish physicians participating in a survey about their work with sick listing were asked about opinions regarding their work as well as needs of knowledge and skills [34]. In that study the most frequent recognised demands were about knowledge and skills in handling of sickness certification, e.g. as regards the assessment of work capacity and optimal length and degree of sickness absence, and information about aspects of the social insurance system.

The physicians in our study reported that they needed more knowledge but also that they needed time to reflect together with colleagues regarding their experiences and thoughts. Thus the question is not only which content to include in continuous medical education aimed to improve sick-listing but also to assess the effect of complementary learning methods including dialogue and shared reflection among physicians.

A limitation of the study is that we did not interview the physicians about their thoughts. However interviews of such a kind may run a risk of being flawed due to recall bias, and that the informant may state what she or he thinks that the interviewer wants to hear. Instead we chose a more indirect approach in three phases and with evaluation from two perspectives. Firstly the participating physicians experienced patient encounters including sick-listing issues. Then they reflected on own experiences and thoughts, both individually and in facilitated small groups together with colleagues. They formulated shared metaphors illustrating challenges in sick-listing. Finally they listed these challenges and suggested actions and stakeholders with a potential to solve the challenges. In order to increase trustworthiness data were gathered both from the action plans formulated by the physicians and from the interviews with the facilitators of the small group meetings. All physicians in this study worked in the same county in Sweden. However it was a big sample representing several disciplines issuing sickness certifications. Of course the reliability can be questioned and in qualitative research the results can never be generalizable to e.g. all physicians working with sick-listing issues. The results however can be transferable and understood even in other contexts and in other groups of physicians.

Due to the qualitative design of this study new and complementary information has been found. This study highlights the fact that the issuing physician is one of several parties in a complex system. This has implications on the choice of educational activities and on professional decision support systems. Thus activities have to support the integration of a range of professional competences and of the awareness of the ambiguity of e.g. the work capacity assessment in every single sickness certification issue. To understand these findings in depth it may be helpful to discuss them in relation to theories of chaos and complexity. A newly reported study by Doll [35] and a corresponding editorial by Mennin [36] criticise the excessive reliance on the use of scientific methods with static representational models to understand health care practice. They argue that developers and educators need to rethink not only the models used, but also the very concept of a model. They prefer the dynamic, ambiguous interplay of complex events that draws more on metaphors and narratives than on models. In this study we started with the physicians’ narratives and own experiences. Then the physicians formed shared metaphors of their common problems and the stakeholders involved. The problems were then mirrored by the experiences of the facilitators. To interpret this complex pattern we realised that we needed a competence framework to understand physicians’ challenges with sick-listing. It was an innovative approach, which worked out quite well. It became obvious that the CanMEDS framework is comprehensive and including rather than excluding. The roles are overlapping and used integrated in the handling of sick-listing issues.

Future studies are needed to increase the understanding of effective learning strategies to develop sufficient skills to effectively and comprehensibly use all the professional competences. We hypothesise that educations shall start out from the participating physicians’ own experiences and include activating learning methods fostering dialogue and reflective capacity in order to improve decision-making when physicians handle sickness certification matters. To perform education together in the health care teams enables opportunities to discuss how to handle organisational problems with sick-listing.

## Conclusions

Physicians performing sickness certification tasks experience a complex variety of challenges. These might be interpreted in relation to a well-established framework of professional competence roles and could only be solved in interaction with medical and non-medical stakeholders. The task is thereby deeply united in the essence of being a physician. Thus, from a public health perspective, educational activities aimed to improve the sick listing process should address all physician competences including identification and interaction with stakeholders, and not just knowledge of social insurance medicine.

## Competing interests

The authors declare that they have no competing interests.

## Authors' contributions

AK designed the study. BA initiated the audit program and coordinated the data collection. BA did the preliminary categorisation of the challenges in the action plans. AK performed the preliminary categorisation of the group interview data. Both authors performed the definitive categorization of the data and drafted the manuscript. BA performed the descriptive statistical analysis. Both authors read and approved the final manuscript.

## Authors information

AK has many years of experience as Senior and Chief Consultant in Internal Medicine and Cardiology. She has a PhD including an educational intervention study in primary care aiming to implement use of evidence-based medicine in routine patient encounters. She holds a position as Senior Lecturer in Medical Education with special emphasize on clinical education at Karolinska Institutet, Stockholm, Sweden.

BA has many years of experience as a specialist in General Practice. She has a PhD including studies on sickness certification. She holds a position as Senior Consultant and Medical expert in Social Insurance Medicine, Stockholm County Council, Sweden.

## Pre-publication history

The pre-publication history for this paper can be accessed here:

http://www.biomedcentral.com/1471-2458/12/702/prepub
